# EffECTively Treating Depression: A Pilot Study Examining Manualized Group CBT as Follow-Up Treatment After ECT

**DOI:** 10.3389/fpsyg.2021.723977

**Published:** 2021-09-03

**Authors:** Luisa Carstens, Corinna Hartling, Sabine Aust, Ann-Kathrin Domke, Anna Stippl, Jan Spies, Eva-Lotta Brakemeier, Malek Bajbouj, Simone Grimm

**Affiliations:** ^1^Berlin Institute of Health, Campus Benjamin Franklin, Charité – Universitätsmedizin Berlin, Corporate Member of Freie Universität Berlin, Humboldt – Universität zu Berlin, Berlin, Germany; ^2^Department for Military Mental Health, German Armed Forces Military Hospital Berlin, Berlin, Germany; ^3^Department of Psychology, Universität Greifswald, Franz-Mehring-Straße, Greifswald, Germany; ^4^Department of Psychiatry, Psychotherapy and Psychosomatics, Psychiatric Hospital, University of Zurich, Zurich, Switzerland; ^5^Department of Psychology, MSB Medical School Berlin, Berlin, Germany

**Keywords:** cognitive behavioral therapy, group therapy, chronic depression, electroconvulsive therapy, cognitive behavioral analysis system of psychotherapy, follow-up treatment

## Abstract

**Background:** There is an urgent need for effective follow-up treatments after acute electroconvulsive therapy (ECT) in depressed patients. Preliminary evidence suggests psychotherapeutic interventions to be a feasible and efficacious follow-up treatment. However, there is a need for research on the long-term usefulness of such psychotherapeutic offers in a naturalistic setting that is more representative of routine clinical practice. Therefore, the aim of the current pilot study was to investigate the effects of a half-open continuous group cognitive behavioral therapy (CBT) with cognitive behavioral analysis system of psychotherapy elements as a follow-up treatment for all ECT patients, regardless of response status after ECT, on reducing depressive symptoms and promoting psychosocial functioning.

**Method:** Group CBT was designed to support patients during the often-difficult transition from inpatient to outpatient treatment. In a non-controlled pilot trial, patients were offered 15weekly sessions of manualized group CBT (called EffECTiv 2.0). The Montgomery-Åsberg Depression Rating Scale was assessed as primary outcome; the Beck Depression Inventory, WHO Quality of Life Questionnaire–BREF, and the Cognitive Emotion Regulation Questionnaire were assessed as secondary outcomes. Measurements took place before individual group start, after individual group end, and 6months after individual group end.

**Results:** During group CBT, Post-ECT symptom reduction was not only maintained but there was a tendency toward a further decrease in depression severity. This reduction could be sustained 6months after end of the group, regardless of response status after ECT treatment. Aspects of quality of life and emotion regulation strategies improved during group CBT, and these improvements were maintained 6months after the end of the group.

**Conclusion:** Even though the interpretability of the results is limited by the small sample and the non-controlled design, they indicate that manualized group CBT with cognitive behavioral analysis system of psychotherapy elements might pose a recommendable follow-up treatment option after acute ECT for depressed patients, regardless of response status after ECT. This approach might not only help to further reduce depressive symptoms and prevent relapse, but also promote long-term psychosocial functioning by improving emotion regulation strategies and psychological quality of life and thus could be considered as a valuable addition to clinical routine after future validation.

## Introduction

Electroconvulsive therapy (ECT) is a very effective treatment option, especially for severe, chronic, and treatment-resistant depression (Kho et al., 2003; Gelenberg et al., 2010). However, a relevant percentage of patients (20–40%) shows no or only partial response (Baldinger et al., 2014) and of those patients who showed initial response, a relevant percentage experiences recurrence of depressive episodes within 6months (Jelovac et al., 2013). These findings underline the importance of an effective follow-up treatment after ECT, to maintain response for those patients who benefited sufficiently and to further support those other patients, who did not benefit sufficiently.

In a previous RCT, ECT responders received 6-month guideline-based antidepressant medication (MED) and were randomly assigned to add-on therapy with cognitive-behavioral group therapy (CBT-arm, *n*=16), add-on therapy with continuation ECT (ECT-arm), or no add-on therapy (MED-arm). The main finding implicates that the cognitive behavioral therapy (CBT) group with elements of cognitive behavioral analysis system of psychotherapy (CBASP; McCullough, 2003) in combination with antidepressants might be an effective follow-up treatment to sustain response after successful ECT in MDD patients (Brakemeier et al., 2014). Also, results of two more studies examining the use of psychotherapy after acute antidepressant ECT treatment were promising: Fenton et al. (2006) found that ECT response could be successfully sustained with a 12-week course of individual cognitive behavioral therapy (CBT) sessions in a sample of six patients. Wilkinson et al. (2017) found that response and remission after ECT could be sustained with a 2-month computer-assisted CBT course in a sample of 12 patients. Due to potential short-term cognitive impairment following acute ECT treatment, psychotherapeutic interventions for these patients are often viewed with skepticism (Fenton et al., 2006). However, these previous findings may provide preliminary evidence that psychotherapeutic interventions after acute ECT could be feasible and effective. Although promising, these previous implementations studies have some limitations in the implementation of the CBT group: Brakemeier et al. (2014) studied a closed CBT group setting with 5–6 patients participating in weekly sessions over a period of 15weeks, led by two experienced psychotherapists. This closed group setting implied a longer waiting period for patients directly after acute ECT treatment, in a period where these patients are especially prone to relapse (Jelovac et al., 2013). In this previous, thoroughly conducted RCT, only patients diagnosed with unipolar depression were included, while patients with bipolar disorders were excluded. However, these patients pose a relevant percentage of patients receiving ECT (Nordenskjöld et al., 2011; Kaster et al., 2021). Most importantly, all three previous studies focused on patients classified as “responders” who benefited from ECT treatment, and disregarded those patients classified as “non-responders” who did not sufficiently benefit from acute ECT treatment. Even though a definition of 50% symptom reduction as “response” is well established (Fenton et al., 2006; Brakemeier et al., 2014; Wilkinson et al., 2017), this dichotomization can be seen as arbitrary categorization, which leads to a situation where especially the depressed patients who did not respond well to ECT, absolutely in need of further treatment, do not receive psychotherapeutic offers.

In general, patients treated with ECT have a long history of illness; they are usually often diagnosed with severe, chronic, or treatment-resistant depression (Schneider et al., 2017). Thus, while some patients respond to ECT and others do not, what these patients share is their psychological burden from severe, often chronic, or treatment-resistant depression with numerous previous treatment attempts with limited outcome. Studies indicate that for this group of patients, psychotherapy, especially CBASP-based interventions, is a recommendable treatment option (Brakemeier et al., 2015; Negt et al., 2016). Some studies suggest that psychotherapeutic interventions, especially when combined with medication, reduce depressive symptoms in the short and long term and promote quality of life and other aspects of social functioning, such as emotion regulation (Keller et al., 2000; Swan et al., 2014; Wiersma et al., 2014; Schramm et al., 2019; Bollmann et al., 2020). Cognitive behavioral analysis system of psychotherapy can be considered as the only psychotherapy specifically developed by McCullough (2003) for the treatment of chronic depression. McCullough (2003) emphasizes social-cognitive skills deficits on the perceptual level (uncoupling of the person-environment interaction) and accordingly suggests that these patients require support particularly concerning their social functioning, specifically concerning emotion regulation and formation of relationships. Thus, CBASP focuses primarily on interpersonal interactions and social problem-solving skills. Group psychotherapy in general has certain advances (cost efficiency, model learning, and universality of suffering; McDermut et al., 2001, Burlingame et al., 2011, Yalom and Leszcz, 2020), and group therapy with CBASP elements seems to be an effective treatment option for both inpatients and outpatients suffering from severe, chronic depression, further emphasizing the focus on social interactions (Brakemeier et al., 2014; Sabaß et al., 2018; Potijk et al., 2020). Thus, while not all patients seem to benefit sufficiently from ECT treatment, it can be assumed that the majority of these patients is suffering from severe, rather chronic depression, which is why group CBT with CBASP elements might be an effective treatment option for ECT responders and non-responders.

Even after depressive symptoms have been successfully reduced and remission has been achieved, psychosocial impairments, such as reduced quality of life and diminished emotion regulation strategies, tend to persist and are associated not only with various socioeconomic risks but also with an increased relapse risk (Scott et al., 2000; Kennedy et al., 2007; Wolkenstein et al., 2014). Thus, an effective treatment option for depressive disorders should not only reduce depressive symptoms but also promote social functioning in the long term, an objective, which is often overlooked and has not been assessed in any of the three previous studies by Fenton et al. (2006), Brakemeier et al. (2014), and Wilkinson et al. (2017).

To the best of the authors’ knowledge, there are a total of three previous studies to date that have investigated the effectiveness and efficacy of psychotherapeutic treatments after acute ECT treatment in severely depressed patients. All three mentioned studies are carefully conducted and provide very promising first findings concerning the effectiveness and efficacy of psychotherapy in this difficult-to-treat population. However, due to the limitations mentioned above (longer waiting period due to closed group setting, exclusion of patients diagnosed with bipolar disorders, as well as exclusion of the so-called non-responders, and omission of psychosocial functioning assessment) these previous implementations allow relatively little assumptions concerning the long-term usefulness of such psychotherapeutic approaches in more naturalistic settings.

Consequently, in a pragmatic approach, the current non-controlled study aimed to examine the effects of a half-open continuous group CBT with CBASP elements as follow-up treatment for all ECT patients regardless of response status after ECT on reducing depressive symptoms and promoting psychosocial functioning.

## Materials and Methods

### Study Design

In the present non-controlled study, we investigated the effects of a half-open group CBT as follow-up treatment after acute ECT. The data presented here are part of a larger ECT study carried out in accordance with the latest version of the Declaration of Helsinki and approved by the Institutional Review Board of Charité – Universitätsmedizin Berlin (clinical trials registration: NCT04159285). Written patient consent was obtained. The group CBT was designed to support patients after acute ECT treatment during the often-difficult transition from inpatient to outpatient treatment. Thus patients could start the group when still hospitalized and were fully discharged or moved to an outpatient clinic during the course of the group.

### Participants

Psychiatric inpatients with a primary diagnosis of a depressive episode in accordance with DSM-5 were offered CBT group participation regardless of response status after an index course of right unilateral ultra-brief ECT at Charité – Universitätsmedizin Berlin. Mean number of administered acute ECT sessions was 12.56 (*SD*=2.21). Due to the half-open group setting, patients were recruited on a rolling basis. Patients self-allocated to the group, which was offered in addition to treatment as usual (TAU; e.g., pharmacological treatment, continuation ECT, individual psychotherapy, or a combination thereof). There were no restrictions regarding TAU; however, medication intake, number of continuation ECT sessions, and individual psychotherapy were documented (see Table 1).

**Table 1:** Demographic and clinical characteristics.

Variable	Overall sample			ECT responders			ECT non-responders			*T*	*df*	Value of *p*
	*M*	*SD*	*n*	*M*	*SD*	*n*	*M*	*SD*	*n*			
Age	48.57	1.78	14	52.50	7.58	8	43.33	14.92	6	−1.51	12	0.157
Education (years)	11.64	1.50	14	11.38	1.52	8	12.00	1.55	6	0.76	12	0.462
Number of psychiatric hospitalization^a^	3	2.18	14	4.38	1.69	8	1.17	1.17	6	−3.98	12	0.002
Number of depressive episodes	4.75	3.20	8	4.75	3.20	8	–	–	–	–	–	–
Duration of current episode (months)^a^	19.25	20.16	8	17.33	22.42	6	25.00	15.56	2	0.44	6	0.677
Pre-ECT MADRS	30.71	7.45	14	29.13	8.98	8	32.83	4.67	6	0.92	12	0.378
T0 MADRS total score	18.07	10.22	14	11.38	5.39	8	27.00	7.95	6	4.40	12	0.001
T1 MADRS total score	13.43	9.91	14	8.88	8.44	8	19.50	8.82	6	2.29	12	0.041
T2 MADRS total score	12.86	13.03	14	6.38	8.92	8	21.50	13.14	6	2.57	12	0.024
Number of ECT sessions	12.57	2.21	14	12.25	2.77	8	13.00	1.26	6	0.61	12	0.551
										***χ*^2^**		**Value of *p***
Gender (F:M)	10:4			5:3			5:1			0.07	1	0.798
Concurrent individual psychotherapy (Y:N)	7:7			5:3			2:4			0.29	1	0.589
Concurrent antidepressant medication (Y:N)	14:0			8:0			6:0					
Concurrent continuation ECT (Y:N)	10:4			8:0			2:4			4.56	1	0.033
Previous psychotherapy (Y:N)	14:0			8:0			6:0					
Previous psychiatric hospitalization (Y:N)	12:2			8:0			4:2			0.98	1	0.321
Previous ECT treatment (Y:N)	2:12			2:6			0:6			0.30	1	0.581
Concurrent acute ECT (Y:N)	3:11			1:7			2:4			0.08	1	0.778
Suicide attempt lifetime (Y:N)	2:12			2:6			0:6			0.30	1	0.581

a *Assumption of equality of error variances violated (Levene’s test: p<0.05)*. *The term “concurrent” refers to treatments received while participating in group therapy.*.

### Intervention

The group manual (called EffECTiv 2.0) is based on the group therapy EffECTive from the Brakemeier et al. (2014) study and the group manual from the PychotherapyPlus study (Bajbouj et al., 2018). A manualized group CBT (called EffECTiv 2.0) with weekly sessions of 100min duration and a maximum number of eight participants were led by two experienced psychotherapists who underwent regular supervision. The CBT-based manual employed a combination of classic cognitive-behavioral interventions, such as mood and activity protocols, and the situational analysis technique described in the CBASP (McCullough, 2003) and by Brakemeier et al. (2021). Patients were offered 15 group sessions, which were framed by two individual sessions before joining the group and one individual session at treatment end. The two individual sessions before joining the group were used to impart psychoeducative information about depressive disorders as well as the group rationale. Moreover, individual goals for the group therapy were determined. The individual session at treatment end was used for a reevaluation of the patients’ goal attainment, appreciation of their progress, and the arrangement of potential further treatment options, such as individual psychotherapy. The manualized group sessions were structured as follows: Short Kiesler’s circle training a warm-up; opening round, situational analysis, including cognitive techniques, and role play; and closing round, including formulation of a “take-home message” to promote generalization and transfer of learning. The Kiesler’s circle model was designed by the American psychologist Donald Kiesler (1983) in order to describe human behavior and the potentially associated social reaction to this behavior. To do so, human behavior can be classified on two domains: friendly hostile and dominant-submissive. The model proposes that certain ways of behaving evoke certain ways of reacting; e.g., acting hostile-dominant might evoke a rather hostile-submissive reaction (and *vice versa*), whereas acting friendly dominant might evoke friendly submissive behavior (and *vice versa*). McCullough assumes that patients suffering from chronic depression often behave rather hostile-submissive, avoiding interactions with others. Goal of the Kiesler’s circle training is to playfully experiment with different ways of behavior and experience the various reactions to these different ways, in order to broaden the patients’ repertoire of behavior (comp. Guhn et al., 2019).

## Assessments

### Primary Outcome

#### Clinician-Rated Depression Severity

##### Montgomery-Åsberg Depression Rating Scale (MADRS)

The MADRS is one of the most commonly used clinical interviews to assess depression severity (Montgomery and Åsberg, 1979). The following 10 depressive symptoms are assessed on a 7-point scale: apparent sadness, reported sadness, inner tension, reduced sleep, reduced appetite, concentration difficulties, lassitude, inability to feel, pessimistic thoughts, and suicidal thoughts. Montgomery-Åsberg Depression Rating Scale assessments took place before the index course of acute ECT (Pre-ECT), as well as after acute ECT and thus before individual group start (T0), after individual group end (T1) and 6months after individual group end (T2). Montgomery-Åsberg Depression Rating Scale reduction of 50% or more during the index course of acute ECT treatment was defined as response (Maust et al., 2012).

### Secondary Outcomes

### Self-Reported Depression Severity

#### Beck Depression Inventory-II (BDI-II)

The BDI-II is a 21-item self-report measure assessing the presence and severity of depressive symptoms, such as sadness, hopelessness, feelings of guilt, fatigue, appetite changes, and reduced sex drive on a 4-point scale (Beck et al., 1996). The BDI-II was assessed before individual group start (T0), after individual group end (T1) and 6months after individual group end (T2).

### Psychosocial Functioning

#### WHO Quality of Life Questionnaire–BREF (WHOQOL-BREF)

The WHOQOL-BREF is a self-report measure assessing the individual global health status (WHOQOL Group, 1998). It consists of 26 items rated on a 5-point scale, assessing four major domains: physical health, psychological health, social relationships, and environment. To enable comparability with the WHOQOL long version, the four domain scores can be transferred into a scale ranging from 0 to 100, with higher scores indicating a more favorable health status. Additionally, the first item addresses “overall individual perception of quality of life,” while the second item refers to “overall individual perception of health.” The WHOQOL-BREF was assessed before individual group start (T0), after individual group end (T1) and 6months after individual group end (T2).

#### Cognitive Emotion Regulation Questionnaire (CERQ)

The CERQ is a self-report measure assessing the following cognitive emotion regulation strategies on a 5-point scale: self-blame, blaming others, rumination, catastrophizing, putting into perspective, acceptance, refocus on planning, positive refocusing, and positive reappraisal (Garnefski and Kraaij, 2007). The higher the respective subscale score, the more the specific coping strategy is employed. The CERQ was assessed before individual group start (T0), after individual group end (T1) and 6months after individual group end (T2).

### Adaptations Due to COVID-19 Pandemic

Due to the COVID-19 pandemic, we stopped conducting group therapy face-to-face in March 2020 and switched to a complete online offer in April 2020, employing a digital healthcare platform.^1^ Prior to the sessions, patients received an email invitation and could then partake *via* video. The original structure of the sessions was maintained except for the warm-up round with a short Kiesler’s circle training, which we found could not be transferred into the online setting in a satisfactory manner.

### Statistical Analyses

All analyses were conducted using SPSS statistical software, version 26 (IBM Corp., United States). *T*-tests for independent samples were used to examine differences between ECT responders and non-responders concerning clinical or demographic variables; chi-squared tests were used to assess differences between categorical variables.

In order to examine the change of clinician-rated depressive symptoms, ANOVAs for repeated measures (Pre-ECT, T0, T1, and T2) were performed for the overall sample and response status was employed as a between-subjects factor. ANOVAs for repeated measures (T0, T1, and T2) were also employed to examine change of quality of life and cognitive emotion regulation strategies during the course of the study.

In order to illustrate the proportion of patients who show a clinically significant change in MADRS score from T0 to T1, the Reliable Change Index (RCI, Jacobson and Truax, 1992) was computed.

Due to limited sample size and our research focus on group CBT effects on all patients receiving acute ECT treatment regardless of response status, separate parametric analyses within the responder and non-responder group were omitted; however, results within the separate groups are depicted in all figures.

All assumptions of the respective tests were satisfied, or it was reasonable to conclude that the tests were robust against the respective violations; thus, only parametric tests were used. Normality of distribution was tested with the Shapiro-Wilk test, equality of error variances was tested with Levene’s test, and Greenhouse-Geisser correction was applied where necessary, as effect size partial *η*^2^ is reported.

## Results

### Clinical and Demographic Data

A total of *n*=27 patients received at least one preliminary psychotherapeutic session, *n*=21 patients started group therapy, and full data sets are available for *n*=14 patients. Of these 14 patients, *n*=2 participated exclusively online (online group as well as online individual sessions framing the group, online follow-up), and *n*=3 changed from face-to-face to online group therapy, with online individual session after group end and online follow-up as well.

**Figure 1:**
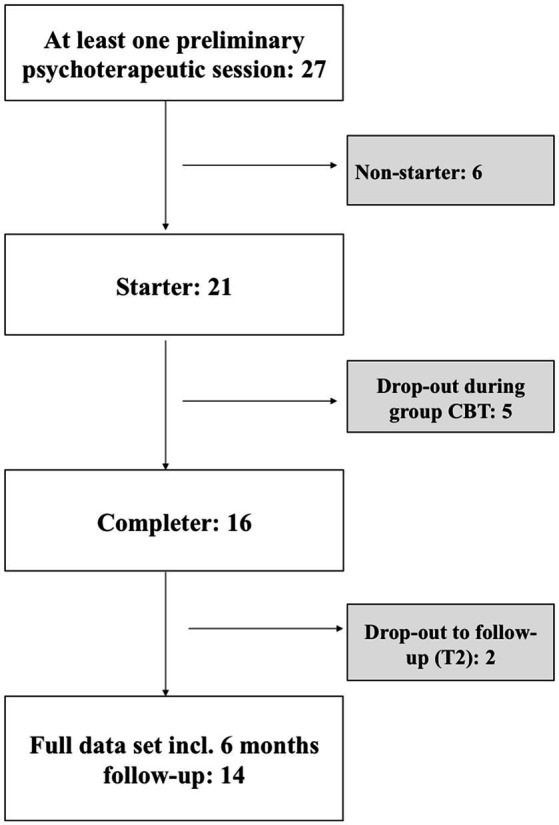
Flowchart of the recruitment process.

For detailed description of the recruitment process, please see Figure 1. Reasons for drop-out during group CBT were time conflict with other appointments (*n*=3) and preference of individual psychotherapy over group therapy (*n*=2). For the 6-month follow-up, *n*=2 patients could not be reached.

Demographic and clinical characteristics for the overall sample, responders, and non-responders are shown in Table 1. All patients received concomitant antidepressant medication. For detailed description of diagnosis type, psychiatric comorbidities, and antidepressant medication, please see Supplementary Tables 1–5, Supplementary Material.

### Primary Outcome

#### Change of Clinician-Rated Depression Severity

**Figure 2:**
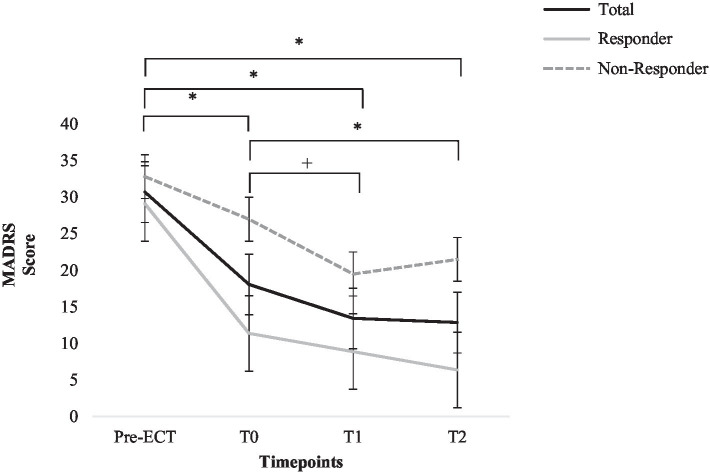
Change in Montgomery-Åsberg Depression Rating Scale score for total sample, responders, and non-responders. Error bars represent standard errors. ^*^=*p*<0.05; +=*p*=0.05 (total sample).

Montgomery-Åsberg Depression Rating Scale scores for the following time points are compared: Pre-ECT, T0, T1, and T2. ANOVAs for repeated measures revealed a significant main effect for time; the MADRS score decreased significantly, *F* (3, 36)=20.03, *p*<0.001, *η*^2^=0.63. A significant between-subjects effect for response was found, *F* (1, 12)=10.81, *p*=0.006, *η*^2^=0.47; per definition, non-responders showed higher MADRS scores (*M*=25.21, *SD*=9.69) than responders (*M*=13.94, *SD*=8.38). No significant time x response interaction effect was found, *p*>0.05, *η*^2^=0.17. Post-hoc tests revealed significant decreases from Pre-ECT (*M*_Pre-ECT_=30.71, *SD*_Pre-ECT_=7.45) to T0, T1, and T2 (*M*_T0_=18.07, *SD*_T0_=10.22, *M*_T1_=13.43, *SD*_T1_=9.91, *M*_T2_=12.86 *SD*_T2_=13.03), *p*<0.001, as well as T0 to T2 (*p*=0.044) and a marginally significant decrease from T0 to T1 (*p*=0.053) in clinician-rated depression severity. See Figure 2 for graphic depiction.

In order to illustrate the proportion of patients who show a clinically significant change in MADRS score from directly after acute ECT treatment (T0) until after group end (T1), the RCI (Jacobson and Truax, 1992) was computed for MADRS change. While 57.1% (*n*=8) of the patients did not show a significant change in MADRS score (RCI between −1.95 and 1.95; Jacobson and Truax, 1992), 35.7% (*n*=5) showed a significant decrease in MADRS score and 7.1% (*n*=1) showed a significant increase in MADRS score.

### Secondary Outcomes

#### Change of Self-Reported Depression Severity

**Figure 3:**
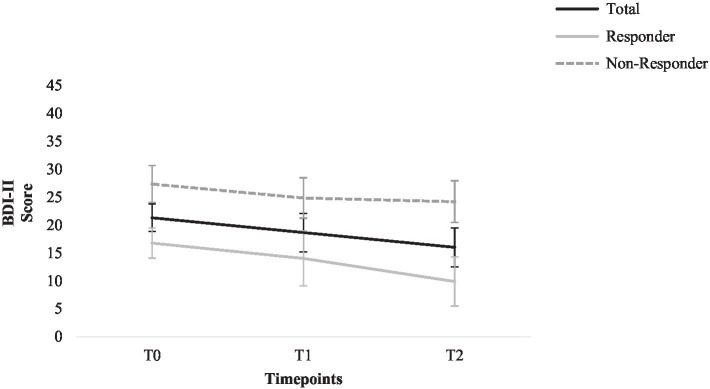
Change in the Beck Depression Inventory-II score for total sample, responders, and non-responders. Error bars represent standard errors.

Beck Depression Inventory-II scores for the following time points are compared: T0, T1, and T2. ANOVAs for repeated measures did not find a significant main effect for time; the BDI-II score did not change significantly from T0 to T2, *F* (2, 26)=2.89, *p*=0.074, *η*^2^=0.18. Post-hoc tests did not find significant changes in self-reported depression severity for all time points *p*>0.05. See Figure 3 for graphic depiction.

### Change of Psychosocial Functioning

#### Change of Quality of Life

**Figure 4:**
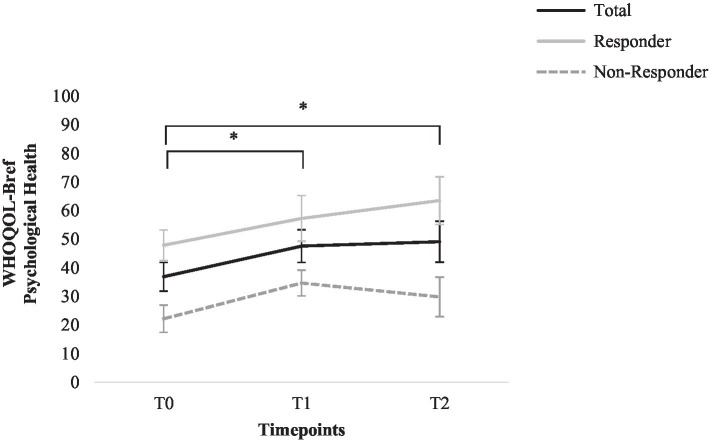
Change in the WHO Quality of Life Questionnaire–BREF psychological health score for total sample, responders, and non-responders. Error bars represent standard errors. ^*^=*p*<0.05 (total sample).

WHO Quality of Life Questionnaire–BREF scores for the following time points are compared: T0, T1, and T2. ANOVAs for repeated measures found a significant main effect for time; a significant increase for the WHOQOL-BREF psychological health scale was found; *F* (2, 26)=4.74, *p*=0.018, *η*^2^=0.27. Post-hoc tests revealed a significant increase from T0 to T1 and T2 (*M*_T0_=36.91, *SD*_T0_=18.69, *M*_T1_=47.62, *SD*_T1_=21.42, *M*_T2_=49.11, *SD*_T2_=26.66) *p<0*.05. No significant effects were found for the WHOQOL-BREF scales physical health, social relationships, and environment (*p*>0.05, *η*^2^=0.01–0.19). See Figure 4 for graphic depiction of change of psychological quality of life; graphic depiction of all other aspects of quality of life can be found in the Supplementary Material (Supplementary Figures 1–3).

### Change of Cognitive Emotion Regulation Strategies

**Figure 5:**
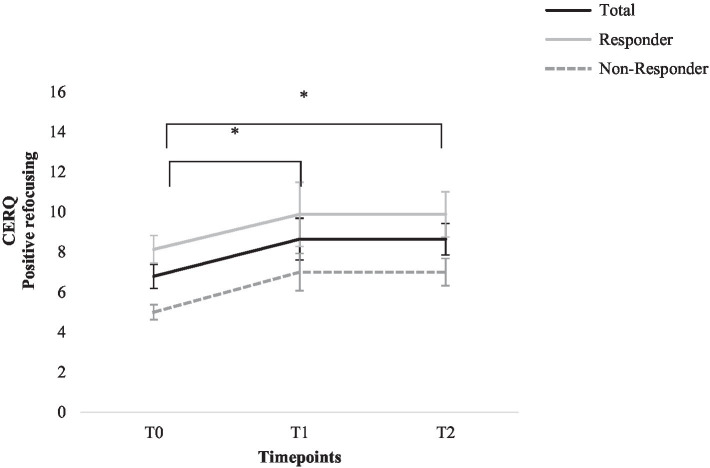
Change in the Cognitive Emotion Regulation Questionnaire positive refocusing score for total sample, responders, and non-responders. Error bars represent standard errors. ^*^=*p*<0.05 (total sample).

Cognitive Emotion Regulation Questionnaire scores for the following time points are compared: T0, T1, and T2. ANOVAs for repeated measures revealed a significant main effect for time; the subscale positive refocusing increased significantly, *F* (2, 26)=4.73, *p*=0.018, *η*^2^=0.27; and post-hoc tests revealed a significant increase from T0 to T1 and T2 (*M*_T0_=6.79, *SD*_T0_=2.23, *M*_T1_=8.64, *SD*_T1_=3.90, *M*_T2_=8.64, *SD*_T2_=2.95), *p*<0.05. No significant effects were found for any of the other adaptive or maladaptive emotion regulation strategies (*p*>0.05, *η*^2^=0.03–0.16). See Figure 5 for graphic depiction of change of positive refocusing; graphic depiction of all other aspects of cognitive emotion regulation strategies can be found in the Supplementary Material (Supplementary Figures 4–11).

## Discussion

### Summary

In this naturalistic feasibility study, we examined the outcome of a half-open CBT group as follow-up treatment after acute antidepressant treatment with ECT. Depression severity and aspects of psychosocial functioning (quality of life and emotion regulation strategies) were assessed during the course of our manualized group CBT with CBASP elements (called EffECTiv 2.0). For the first time, all patients regardless of response status after acute ECT were offered group participation. Assessments took place Pre-ECT, before individual group start (T0), after individual group end (T1), and 6months after individual group end (T2).

As primary outcome, change in clinician-rated depression severity (MADRS) was assessed. During the course of the study, ANOVAs revealed a significantly decreased clinician-rated depression severity (MADRS). Montgomery-Åsberg Depression Rating Scale score decreased significantly during ECT treatment, and there was a trend toward further decreased depression severity during group CBT after ECT for all patients. Six months after group end, MADRS scores were still significantly lower than Pre-ECT treatment and Pre-group start; thus, symptom improvement after group CBT could be successfully maintained. Accordingly, the RCI indicated a clinically significant further decrease in depression severity during group CBT for 35.7% of the patients, while 57.1% of the patients maintained their Post-ECT symptom improvement and only 7.1% showed a significant increase in depression severity.

As secondary outcomes, self-reported depression severity and aspects of psychosocial functioning were assessed. Considering self-reported depression severity measured with the BDI-II, there was a slight decrease; however, the effects were not significant.

As a long-term effective treatment should not only decrease depressive symptoms but also increase psychosocial functioning (Scott et al., 2000; Kennedy et al., 2007), we also assessed quality of life and cognitive emotion regulation strategies. During group CBT, psychological quality of life was significantly increased and could be maintained 6months after group end. For other aspects of quality of live measured with the WHOQOL-BREF (physical health, social relationships, and environment), no significant effects could be found.

Regarding cognitive emotion regulation strategies assessed with the CERQ, a significant increase for the adaptive emotion regulation strategy “positive refocusing” was found during group therapy, with a large effect size. The increase could be successfully maintained 6months after group end. For none of the other adaptive or maladaptive strategies, a significant change was found.

### Comparison With Other Findings

Response rate after acute ECT (57%) was relatively low compared to previous studies. This might be due to clinical characteristics of our sample, such as the relatively low percentage of patients with psychotic features, diagnosis polarity, and the exclusion of patients diagnosed with schizoaffective disorders (Nordenskjöld et al., 2011; Pinna et al., 2018) as well as technical differences, such as electrode placement, dosage, seizure duration, and utilized anesthetic (Merkl et al., 2009).

The successful maintenance of symptom improvement after ECT with CBT-based interventions is in accordance with previous findings (Fenton et al., 2006; Brakemeier et al., 2014). Additionally, we found not only maintenance effects, but also a trend toward further reduction in depression severity in both ECT responders and non-responders during group CBT, which could be maintained 6months after group end. Accordingly, Brakemeier et al. (2014), Fenton et al. (2006), and Wilkinson et al. (2017) also reported a trend toward further symptom improvement after CBT in ECT responders. In our CBT sample, only 7.1% of the patients experienced a significant worsening of depressive symptoms, while the majority of patients either showed maintained or decreased depression severity. In general, severely depressed patients treated with ECT are prone to relapse, even after a successful course of ECT treatment. Relapse and rehospitalization rates within six to 12months after acute ECT are high (up to 66%, Cakir and Caglar, 2017), and relapse rates remain high even with continuation ECT or concomitant pharmacological treatment (34–47%; Sackeim et al., 2001; Jelovac et al., 2013; Brakemeier et al., 2014; Verwijk et al., 2015). In our sample, only one patient was rehospitalized during the course of the study and 92.8% of the patients either maintained or improved with regard to their depressive symptoms.

While promising outcomes were found for clinician-rated depression severity, no significant effects were found with regard to self-reported depression severity measured with the BDI-II. However, self-reported depression severity Post-ECT was maintained during the course of the study, in accordance with findings from Fenton et al. (2006). Discrepancies between clinician and self-reported measures of depression severity have been described in the literature (Uher et al., 2008), and a large meta-analysis by Cuijpers et al. (2010) reported that the use of self-reported measures tends to result in smaller effects in psychotherapy studies. As a possible explanation, the authors discuss a greater sensitivity of change in clinician-rated measures, self-report measures being more conservative, or measures assessing different symptoms of depression.

In accordance with previous findings, we found increased psychological quality of life in depressed patients after CBT treatment (Lenz and Demal, 2000; Swan et al., 2009). Sabaß et al. (2018) also reported increased psychological quality of life in chronically depressed inpatients treated with CBASP group therapy. However, Sabaß et al. (2018) additionally reported increased quality of life concerning physical health, social relationships, and environment, which we did not find in our study. Possible explanations for this discrepancy could be Sabaß et al.’s larger sample size and their general study design examining inpatient CBASP therapy. This implies a higher frequency of psychotherapeutic interventions than we examined. Moreover, this inpatient sample might lead to a more positive assessment of other aspects of quality of life due to patients’ protected environment with less potential for everyday life challenges.

Concerning cognitive emotion regulation strategies, patients reported an increased use of the adaptive strategy “positive refocusing.” While previous research proposed the assessment of emotion regulation strategies and especially the utilization of the CERQ in psychotherapy studies with depressed patients (Garnefski and Kraaij, 2007; Lei et al., 2014), no previous studies examining the association between CBT-based interventions and the CERQ could be found. Two studies examining emotion regulation strategies in depressed patients found a significant increase in cognitive reappraisal measured with the ERQ (Gross and John, 2003) after CBT treatment (Forkmann et al., 2014; Bollmann et al., 2020), which can be linked to our findings.

### Implications

Our findings imply an effect of group CBT as follow-up treatment after acute ECT. Symptom improvement was not only maintained, but depression severity was further reduced and psychological quality of life and the use of adaptive cognitive emotion regulation strategies increased in both ECT responders and non-responders. Considering the high relapse rates and the substantial proportion of patients who only benefit partially from ECT treatment (Baldinger et al., 2014), these findings might be of clinical relevance, as they might propose an effective follow-up treatment option for a difficult-to-treat patient population with a long history of suffering. The increased use of adaptive cognitive emotion regulation strategies seems especially relevant, as deficits in emotion regulation tend to persist even after remission of depressive symptoms and might pose a risk factor for the recurrence of depressive symptoms (Ehring et al., 2008). Likewise, quality of life also seems to be reduced in subsyndromal as well as remitted patients (Angermeyer et al., 2002; da Silva Lima and de Almeida Fleck, 2007; Shimizu et al., 2013), while the increased psychological quality of life and reduced mental pain are one of the most important treatment outcomes according to self-reports of depressed patients (Chevance et al., 2020) and thus should be targeted in a comprehensive antidepressant treatment. In general, psychotherapeutic interventions tend to show sustainable effects even after treatment end which makes them an especially appealing follow-up treatment (Hensley et al., 2004; Schramm et al., 2019; Potijk et al., 2020).

### Limitations and Suggestions for Future Research

A number of limitations need to be mentioned. First of all, considering the small sample size, our pilot study has to be regarded as underpowered and generalizability is limited. Due to the small sample size, Bonferroni corrections for post-hoc tests were omitted (Nakagawa, 2004), which might have led to false positive results. Furthermore, due to the naturalistic setting and the absence of a thoroughly assessed control group, potential confounding effects of continuation ECT, antidepressant medication, or individual psychotherapy cannot be ruled out and general conclusions are restricted. However, this naturalistic setting can also be regarded as an advantage, as it reflects clinical reality and thus might allow assumptions concerning the usefulness of this group CBT concept in clinical routine. Due to the COVID-19 pandemic, the study design was adapted and group therapy was carried out online. Of our total sample, five patients participated partially or exclusively online, which might confound findings. However, general mental health, psychosocial functioning, and wellbeing as well as depressive symptoms are negatively affected by the COVID-19 pandemic, especially in populations with preexisting psychiatric disorders, underlining the increased vulnerability of our sample (Pfefferbaum and North, 2020; Salari et al., 2020; Vindegaard and Benros, 2020). Hence, our promising results despite a worldwide pandemic can be regarded as especially encouraging and underline the potential use of group CBT for these patients. To allow for robust assumptions, future studies with larger sample sizes and control groups are needed. However, hesitations concerning psychotherapeutic interventions directly after acute ECT still persist, especially emphasizing cognitive side effects after acute ECT (McClintock et al., 2011). Thus, psychoeducative measures about the potential of these interventions addressing clinicians and patients alike might be helpful as well as accompanying neuropsychological assessments and qualitative surveys of patients’ perception of these psychotherapeutic interventions. Moreover, as ultra-brief and RUL ECT treatment is associated with less cognitive side effects (Merkl et al., 2009), an adaption of ECT protocols could be considered when including psychotherapy into the treatment strategy. Less cognitive side effects might enable and motivate more patients to participate in a CBT offer. Our findings indicate that responders and non-responder might both benefit from CBT after acute ECT treatment. However, due to our limited sample size, sub-sample analyses separately for responders and non-responders were omitted. Thus, the future studies with a larger sample size allowing for sub-sample analyses might prove enlightening. Accordingly, an additional individualization of the group manual (EffECTiv 2.0) for this special population, including responders and non-responders, could be interesting. For example, emotion regulation skills could be added to the manual or additional flexible support for patients in need could be offered. This additional support could be provided either during the group session (as an index patient) or on the basis of additional individual sessions.

## Conclusion

To the authors’ best knowledge, this is the first time effects of group CBT as follow-up treatment after ECT was examined regardless of ECT response status. In this pilot study, symptom improvement after ECT treatment was not only maintained but there was a tendency toward further reductions in depression severity, which could be sustained 6months after group end. For the first time, the changes in psychosocial functioning were assessed as well and results suggest favorable effects on cognitive emotion regulation strategies and psychological quality of life that could also be maintained 6months after group end. Thus, our findings provide preliminary evidence that after future validation, group CBT with CBASP elements might provide a useful addition to clinical routine as follow-up treatment for all patients after antidepressant ECT treatment, regardless of response status.

## Data Availability Statement

The raw data supporting the conclusions of this article will be made available by the authors, without undue reservation.

## Ethics Statement

The studies involving human participants were reviewed and approved by the Institutional Review Board of the Charité – Universitätsmedizin Berlin. The patients/participants provided their written informed consent to participate in this study.

## Author Contributions

LC made substantial contributions to the conception and design of the work; the acquisition, analysis, and interpretation of data; and wrote the manuscript. CH made substantial contributions to the conception and design of the work, and the acquisition of data. SA made substantial contributions to the conception and design of the work. A-KD and AS made substantial contributions to the acquisition, analysis, and interpretation of data. JS made substantial contributions to conception of the study and the psychotherapeutic concept. The psychotherapeutic concept was based on the previous work from E-LB who was also involved in drafting the manuscript. MB made substantial contributions to conception and design of the study. SG made substantial contributions to conception and design of the study as well as analysis and interpretation of data and has been involved in drafting the manuscript. All authors revised the work critically for important intellectual content, approved the final version to be published, and agreed to be accountable for all aspects of the work in ensuring that questions related to the accuracy or integrity of any part of the work are appropriately investigated and resolved.

## Conflict of Interest

The authors declare that the research was conducted in the absence of any commercial or financial relationships that could be construed as a potential conflict of interest.

## Publisher’s Note

Publisher’s Note: All claims expressed in this article are solely those of the authors and do not necessarily represent those of their affiliated organizations, or those of the publisher, the editors and the reviewers. Any product that may be evaluated in this article, or claim that may be made by its manufacturer, is not guaranteed or endorsed by the publisher.

## Footnotes

^1^ https://www.comjoodoc.de/en/home-eng/
