# Supplementary material for: EffECTively Treating Depression: A Pilot Study Examining Manualized Group CBT as Follow-Up Treatment After ECT
Source: Front Psychol. 2021 Sep 3;12:723977. doi: 10.3389/fpsyg.2021.723977 (PMC8446269; doi:10.3389/fpsyg.2021.723977)
Supplement: Supplementary file 1 [file Data_Sheet_1.docx]

Supplementary Material

Supplementary Table 1

*Frequencies of Clinical Characteristics*

| Variable | Frequencies |
| --- | --- |
| Diagnosis Polarity | 92.9% unipolar (13)  7.1% bipolar (1) |
| Diagnosis | - 7.1% F31.3: Bipolar affective disorder, current episode mild or moderate depression (1) - 21.4% F32.1: Moderate depressive episode (3) - 7.1% F33.1: Recurrent depressive disorder, current episode moderate (1) - 50.0% F33.2: Recurrent depressive disorder, current episode severe without psychotic symptoms (7) - 7.1% F33.3: Recurrent depressive disorder, current episode severe with psychotic symptoms (1) - 7.1% F34.1: Dysthymia (1) |
| Psychiatric comorbidity | 57.1% yes (8)  42.9% no (6) |
|  | 21.4% F30-F39: affective disorder (3)  7.1% F40-F48: anxiety, stress-related, or somatoform disorders (1)  14.3% F50.-: eating disorders (2)  21.4% F60-F69: personality disorders (3) |
| Somatic disease | 50.0% yes (7) 50.0% no (7) |
|  | 7.1% A00-B99: infectious or parasitic (1)  7.1% C00-D48: Neoplasms (1)  7.1% D50-D90: of the blood and blood-forming organs and certain disorders involving the immune mechanism (1)  35.7% E00-E89: endocrine, nutritional or metabolic (5)  14.3% G00-G99: of the nervous system (2)  7.1% H00-H95: of the eye or ear or nose (1)  35.7% I00-I99: of the circulatory system and heart (5)  7.1% L00-L99: of the skin or musculoskeletal system (1)  14.3% M00-M99: of the musculoskeletal system and connective tissue (2) 21.4% Z00-Z99: presence of factors influencing health status (3) |
| *Notes*. Subgroups of psychiatric and somatic comorbidities refer to ICD-10 classifications. Percentage scores might not add up to exactly 100 % due to patients having more than one diagnosis. | |

Supplementary Table 2

*Frequencies of Antidepressant Medication Use at baseline (T0)*

| Variable | Frequencies |
| --- | --- |
| Antidepressants (ADs) | 00.0% none (0)  100.0% ADs (14):  50.0% SSRIs (7)  42.9% SSNRI (6)  00.0% SNRI (0) 21.4% NDRIs (3) 00.0% SARIs (0) 14.3% TCAs (2)  7.1% TeCAs (1)  00.0% MAOI (0)  7.1% Melatonergic agonist (1) |
| Other psychiatric medication | 64.3% none (9)  35.7% others (5):  28.6% antipsychotics (4)  14.3% mood stabilizer (2)  00.0% benzodiazepines (0) |
| Change in Medication  (baseline to T1) | 57.1% no (8)  42.9% yes (6) |
| Change in ADs  (baseline to T1) | 57.1% no change (8)  28.6% change in type (4):  00.0% switch to different type (0)  21.4% cessation (3)  7.1% start of new AD (1)  14.3% change in dosage (2):  7.1% increase (1)  7.1% reduction (1) |
| Change in other psychiatric medication  (baseline to T1) | 78.6% no change (11)  14.3% change in type (2):  00.0% switch to different type (0)  14.3% cessation (2)  00.0% start of new medication (0)  7.1% change in dosage (1):  7.1% increase (1)  00.0% reduction (0) |
| *Notes*. SSRIs= selective Serotonin-Reuptake-Inhibitors. SSNRIs= selective Serotonin–norepinephrine reuptake inhibitors. Selective norepinephrine reuptake inhibitor. NDRIs= Norepinephrine-dopamine reuptake inhibitors. SARIs= Serotonin antagonist and reuptake inhibitors. TCAs= Tricyclic antidepressants. TeCAs= Tetracyclic antidepressants. MAOIs= Monoamine oxidase inhibitors Percentage scores might not add up to exactly 100 % due to patients taking more than one psychiatric medication. | |

Supplementary Table 3

*Frequencies of Antidepressant Medication Use at T1*

| Variable | Frequencies |
| --- | --- |
| Antidepressants (ADs) | 00.0% none (0)  100.0% ADs (14):  35.7% SSRIs (5)  50.0% SSNRI (7)  7.1% SNRI (1) 21.4% NDRIs (3) 14.3% SARIs (2) 14.3% TCAs (2)  7.1% TeCAs (1)  00.0% MAOIs (0)  7.1% Melatonergic agonist (1) |
| Other psychiatric medication | 85.7% none (11)  21.4% others (3):  21.4% antipsychotics (3)  7.1% mood stabilizer (1)  00.0% benzodiazepines (0) |
| Change in Medication  (T1 to T2) | 28.6% no (4)  71.4% yes (10) |
| Change in ADs  (T1 to T2) | 28.6% no change (4)  35.7% change in type (5):  00.0% switch to different type (0)  28.6% cessation (4)  7.1% start of new AD (1)  28.6% change in dosage (4):  14.3% increase (2)  14.3% reduction (2) |
| Change in other psychiatric medication  (T1 to T2) | 85.7% no change (12)  7.1% change in type (1):  00.0% switch to different type (0)  7.1% cessation (1)  00.0% start of new medication (0)  7.1% change in dosage (1):  00.0% increase (0)  7.1% reduction (1) |
| *Notes*. SSRIs= selective Serotonin-Reuptake-Inhibitors. SSNRIs= selective Serotonin–norepinephrine reuptake inhibitors. Selective norepinephrine reuptake inhibitor. NDRIs= Norepinephrine-dopamine reuptake inhibitors. SARIs= Serotonin antagonist and reuptake inhibitors. TCAs= Tricyclic antidepressants. TeCAs= Tetracyclic antidepressants. MAOIs= Monoamine oxidase inhibitors Percentage scores might not add up to exactly 100 % due to patients taking more than one psychiatric medication. | |

Supplementary Table 4

*Frequencies of Antidepressant Medication Use at T2*

| Variable | Frequencies |
| --- | --- |
| Antidepressants (ADs) | 14.3% none (2)  85.7% ADs (12):  21.4% SSRIs (3)  35.7% SSNRI (5)  14.3% SNRI (2) 21.4% NDRIs (3) 00.0% SARIs (0) 14.3% TCAs (2)  7.1% TeCAs (1)  00.0% MAOIs (0)  7.1% Melatonergic agonist (1) |
| Other psychiatric medication | 85.7% none (12)  14.3% others (2):  14.3% antipsychotics (2)  7.1% mood stabilizer (1)  00.0% benzodiazepines (0) |
| *Notes*. SSRIs= selective Serotonin-Reuptake-Inhibitors. SSNRIs= selective Serotonin–norepinephrine reuptake inhibitors. Selective norepinephrine reuptake inhibitor. NDRIs= Norepinephrine-dopamine reuptake inhibitors. SARIs= Serotonin antagonist and reuptake inhibitors. TCAs= Tricyclic antidepressants. TeCAs= Tetracyclic antidepressants. MAOIs= Monoamine oxidase inhibitors Percentage scores might not add up to exactly 100 % due to patients taking more than one psychiatric medication. | |

Supplementary Table 5

*Frequencies of Medication Change Overall*

| Variable | Frequencies |
| --- | --- |
|  |  |
| Change in Medication | 7.1% no (1)  92.9% yes (13) |
| Change in ADs | 14.3% no change (2)  42.9% change in type (6):  00.0% switch to different type (0)  35.7% cessation (5)  7.1% start of new AD (1)  42.9% change in dosage (6):  14.3% increase (2)  50.0% reduction (4) |
| Change in other psychiatric medication | 64.3% no change (9)  28.6% change in type (4):  00.0% switch to different type (0)  28.6% cessation (4)  00.0% start of new medication (0)  7.1% change in dosage (1):  00.0% increase (0)  7.1% reduction (1) |
| Percentage scores might not add up to exactly 100 % due to patients taking more than one psychiatric medication. | |

**Change of psychosocial functioning**

**Quality of life.**


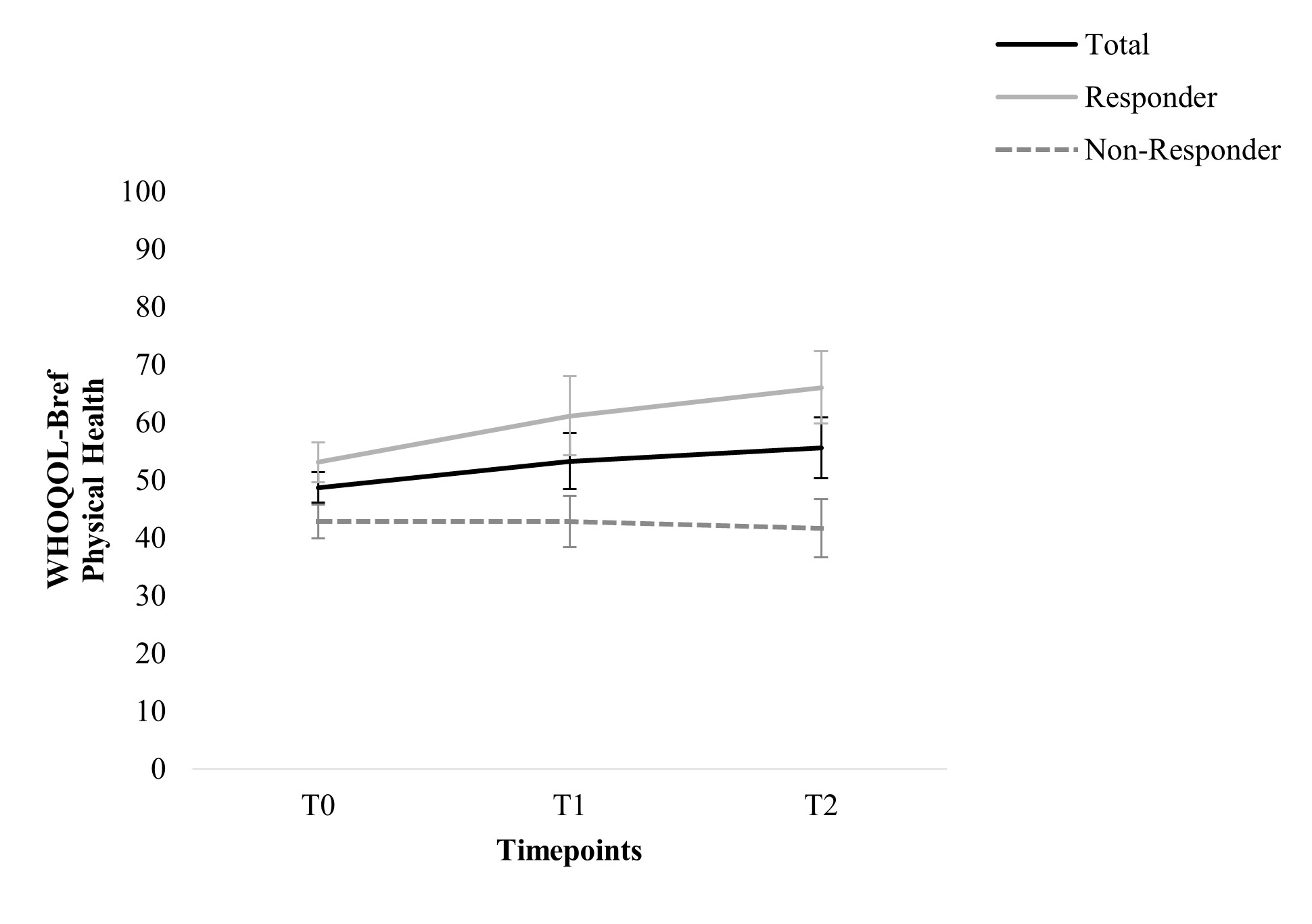


**Supplementary Figure 1:** Change of physical health score for total sample, responders and non-responders. Error bars represent standard errors.


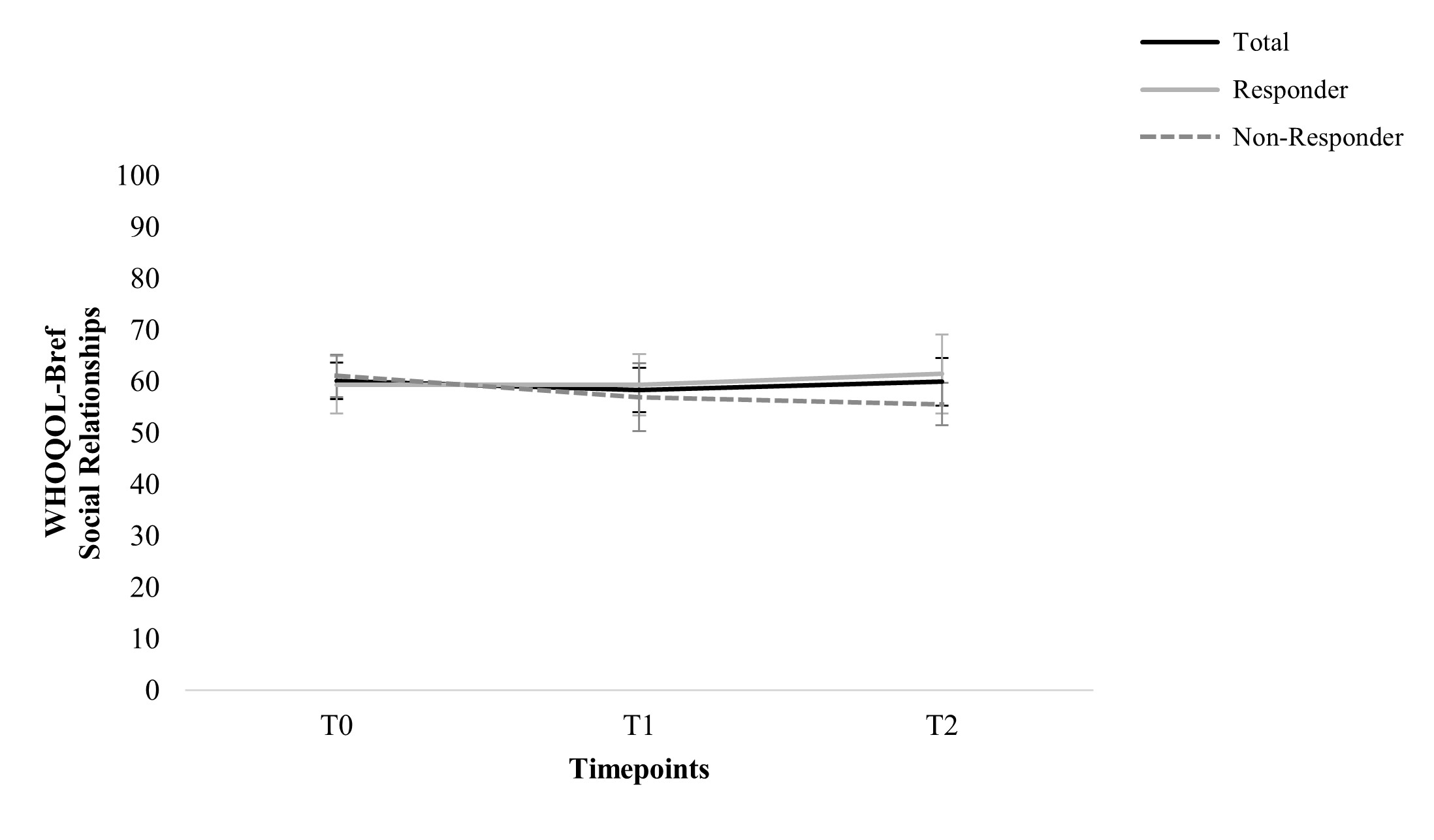


**Supplementary Figure 2:** Change of social relationships score for total sample, responders and non-responders. Error bars represent standard errors.


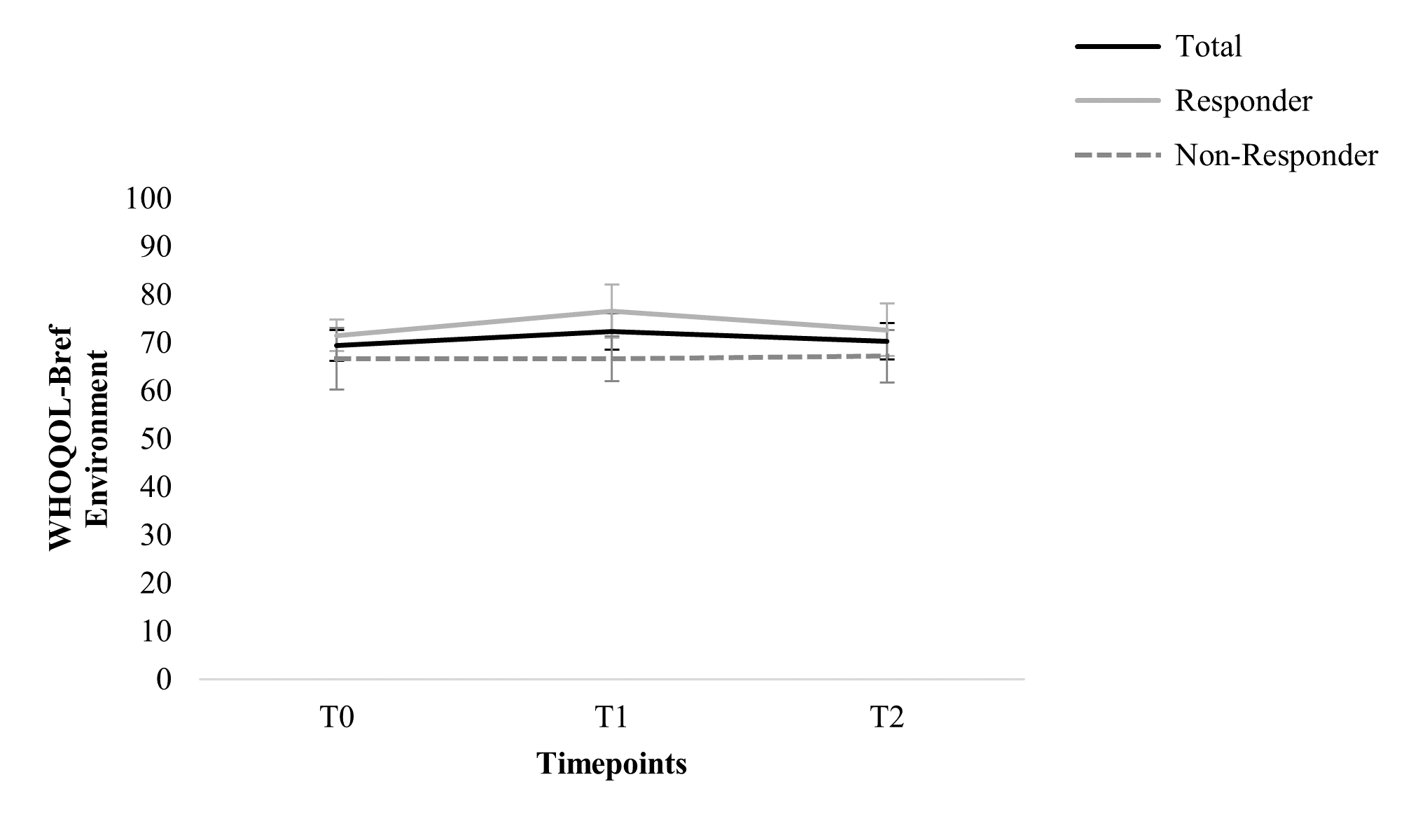


**Supplementary Figure 3:** Change of environment score for total sample, responders and non-responders. Error bars represent standard errors.

**Cognitive Emotion Regulation Strategies.**

**Supplementary Figure 4:** Change of self-blame score for total sample, responders and non-responders. Error bars represent standard errors.

**Supplementary Figure 5:** Change of acceptance score for total sample, responders and non-responders. Error bars represent standard errors.

**Supplementary Figure 6:** Change of rumination score for total sample, responders and non-responders. Error bars represent standard errors.

**Supplementary Figure 7:** Change of refocus on planning score for total sample, responders and non-responders. Error bars represent standard errors.

**Supplementary Figure 8:** Change of positive reappraisal score for total sample, responders and non-responders. Error bars represent standard errors.

**Supplementary Figure 9:** Change of putting into perspective score for total sample, responders and non-responders. Error bars represent standard errors.

**Supplementary Figure 10:** Change of catastrophizing score for total sample, responders and non-responders. Error bars represent standard errors.

**Supplementary Figure 11:** Change of blaming others score for total sample, responders and non-responders. Error bars represent standard errors.
